# A prediction model of compressor with variable-geometry diffuser based on elliptic equation and partial least squares

**DOI:** 10.1098/rsos.171468

**Published:** 2018-01-24

**Authors:** Xu Li, Chuanlei Yang, Yinyan Wang, Hechun Wang

**Affiliations:** College of Power and Energy Engineering, Harbin Engineering University, Heilongjiang Sheng, People's Republic of China

**Keywords:** diesel engine, variable geometry compressor, performance prediction, PLS model

## Abstract

To achieve a much more extensive intake air flow range of the diesel engine, a variable-geometry compressor (VGC) is introduced into a turbocharged diesel engine. However, due to the variable diffuser vane angle (DVA), the prediction for the performance of the VGC becomes more difficult than for a normal compressor. In the present study, a prediction model comprising an elliptical equation and a PLS (partial least-squares) model was proposed to predict the performance of the VGC. The speed lines of the pressure ratio map and the efficiency map were fitted with the elliptical equation, and the coefficients of the elliptical equation were introduced into the PLS model to build the polynomial relationship between the coefficients and the relative speed, the DVA. Further, the maximal order of the polynomial was investigated in detail to reduce the number of sub-coefficients and achieve acceptable fit accuracy simultaneously. The prediction model was validated with sample data and in order to present the superiority of compressor performance prediction, the prediction results of this model were compared with those of the look-up table and back-propagation neural networks (BPNNs). The validation and comparison results show that the prediction accuracy of the new developed model is acceptable, and this model is much more suitable than the look-up table and the BPNN methods under the same condition in VGC performance prediction. Moreover, the new developed prediction model provides a novel and effective prediction solution for the VGC and can be used to improve the accuracy of the thermodynamic model for turbocharged diesel engines in the future.

## 1. Introduction

The turbocharger is one of the most vital parts in a modern diesel engine. It allows increase in engine power density through the downsizing concept [1–5], reducing fuel consumption. The compressor, as an important component of the turbocharger, is used to compress the intake air. Air is compressed in the compressor in order to increase its density in the diesel engine. The compressor is powered by a turbine and the turbine is powered by the exhaust gas of the diesel engine. However, when the diesel engine is operated under a low-power condition, the compressor loses the ability to provide adequate air supply with proper pressure for the diesel engine [6]. To solve this problem, the variable-geometry compressor (VGC [7–10], including a variable inlet guide vane [11–13] and variable-geometry diffuser [14–16]), is introduced into the turbocharger in the diesel engine. With the VGC, the operation range and the efficiency of compressor are expanded extensively, particularly under low mass flow rate and high pressure ratio conditions [17]. Therefore, the performance of the diesel engine can be improved greatly with the VGC, especially at low power and low speed.

It is obvious that the performance of the compressor plays a vital role in predicting and diagnosing the performance of the diesel engine [18]. The quality of the compressor performance map is essential for evaluating the accuracy of diesel engine performance and the diagnostic model. Typically, the behaviour of the compressor is represented by the performance map which is supplied by the original equipment manufacturer. The map can also be determined by stream line curvature and computational fluid dynamics (CFD) [19] if the detailed geometry of the compressor is known. In general, considering the complicated features of the compressor, experimental study may be the most suitable method to obtain the compressor performance map, under various operating and environmental conditions. However, because of the huge cost and potential hazards of the compressor test, experimental study is not suitable for compressor performance, especially with the VGC, with a more variable diffuser vane angle (DVA) than the common compressor. To improve the accuracy of diesel engine performance prediction, many researchers explored alternative methods for obtaining the compressor map.

As the most popular method for representing the characteristics of the compressor, the look-up table uses the experimental compressor mass flow rate and efficiency maps [20]. The map is represented by the forms of lines of constant reduced speed and efficiency. The reduced speed and efficiency of each point which is not located at reduced speed and efficiency lines are interpolated and extrapolated by the experimental maps linearly [21]. As we know, the compressor map has a strong nonlinear relationship between its parameters (i.e. reduced speed, pressure ratio, mass flow rate and efficiency). Therefore, the prediction accuracy of the compressor map using look-up tables is dependent on the quantity and quality of experimental data. The look-up tables lose their ability for either interpolating or extrapolating the reduced speed lines and efficiency lines under acceptable accuracy with few experimental data.

Another method used is shifting and scaling the shape of a similar compressor map through polynomial equations such that it matches the targeted compressor's performance. This method was introduced by Kong *et al*. [22]. They assumed that the shape of the implemented map is very similar to the actual map. To determine the coefficients of the polynomials, Kong *et al.* incorporated the Genetic Algorithms into this identification method [23]. Li *et al*. [24] suggested a unique set of scaling coefficients for each line of constant speed and efficiency to capture nonlinear effects. This method is more accurate than the traditional scaling methods.

Some researchers focus on the intelligent algorithm. As an effective data-based modelling method, the artificial neural network (ANN) is widely used in many areas [25–30] because of its capability of dealing with nonlinear processes and storing a massive amount of experimental information. Theoretically, the ANN can approximate any nonlinear model and develop the relationship between input and output variables involved in a physical process without considering the underlying physical process [26–29]. Yu *et al*. introduced back-propagation neural networks (BPNNs) [28] into predicting the performance of the compressor, which is an improved ANN method. To provide good prediction effect of the compressor, multi-layer perception approach is used in BPNNs. Although the BPNN is effective in interpolation prediction of the compressor map, its predictive accuracy is poor in extrapolation prediction of the compressor map [31]. Moreover, it is important to point out that a large number of experimental data are necessary to sufficiently train BPNNs to get high prediction accuracy and calculation stability. As an advanced BPNN, the extreme learning machine has a strong performance prediction ability in the biodiesel engine. Biodiesel has great advantages in emission and environmental affinity, but it also has the disadvantages of lack of motivation and excessive emission of nitrogen oxides. Therefore, it is necessary to study the proportion of biodiesel and diesel in order to obtain the optimal emission characteristics and dynamic characteristics. Sebayang [32] and Silitonga [33] use the different extreme learning machine to get the best proportion of biodiesel in the gasoline engine and the turbocharged diesel engine. However, the extreme learning machine also needs large data, which is inadequate in VGC performance prediction.

The prediction accuracy of these methods depends on the quality and quantity of compressor data supplied by compressor manufacturers or compressor users, especially with the look-up table method. However, because of secrecy and other reasons, manufacturers only supply the design-condition points instead of the entire performance map, which makes these methods difficult to meet the requirement of compressor performance prediction.

When the inlet flow area or the diffuser inlet flow area of the VGC is changed by rotating inlet vanes or diffuser vanes, respectively, the performance of the VGC is changed visibly. It means the original maps, namely the performance maps of the compressor with stationary inlet vanes and diffuser vanes, are not suitable for predicting the performance of the changed compressor. A new rotation angle means a new compressor, which proposes a great challenge for researchers to predict the performance of the VGC. Despite this challenge, some endeavours have been made to meet the requirement of predicting performance of the VGC because of its great benefit. Xue *et al*. used the flow loss model to predict the performance of the VGC [34]. Through considering the enthalpy loss of each component of the compressor, and modifying the enthalpy loss model based on the inlet vanes’ rotational angle, they predicted the performance at random inlet vane angle, speed and mass flow rate. However, the accuracy of this method relied on the selected enthalpy loss model and detailed geometrical data, which limits the scope of its application. Ying *et al*. [35–38] introduced partial least squares into predicting the performance of the VGC. Using this method, the performance is predicted with high accuracy, without caring about the detailed geometrical structure and needing less performance data than other methods. However, in order to get suitable accuracy, the order of the polynomial should be very high. This means the number of coefficients is enormous, even more than 300, which is not suitable for rapid performance prediction and online control.

In this paper, a compressor map generation method for improving the accuracy of compressor performance prediction is developed. Different from previous methods, the characteristic lines of the compressor are expressed as mathematical equations of an ellipse with a fixed centre and rotational coordinates. Then the partial least-squares method is employed to decrease the huge number of coefficients caused by the characteristic lines of the VGC. Next, the prediction of VGC performance has been done by the proposed methods, the look-up table method and BPNNs. Finally, the proposed methods and contrastive methods are compared with regard to accuracy, complexity and computational time of extrapolation, interpolation and predicting an overall performance map of a constant-angle VGC.

## 2. Methodology

The compressor performance map used in the diesel engine thermodynamic models is presented as four key parameters: pressure ratio *π_C_*, the corrected mass flow rate *m_C_*, the isentropic efficiency *η*_C_ and corrected rational speed *N*. For the VGC, an additional parameter, the rational angle of diffuser vanes *α*, is introduced. The objective of map generation methods is to obtain mathematical expression that could capture the shape of the performance map accurately. In the VGC, this is performed by relating the pressure ratio *π_C_* and the isentropic efficiency *η*_C_ with the corrected mass flow rate *m_C_*, the corrected rational speed *N* and the rational angle of diffuser vanes *α*. It means $\pi_{C}=f(m_{C},N,\alpha )$ and $\eta_{C}=g(m_{C},N,\alpha )$. The detailed description of the proposed methods will be demonstrated in §2.2.

### 2.1. The original map and VGC map

**Figure 1.**
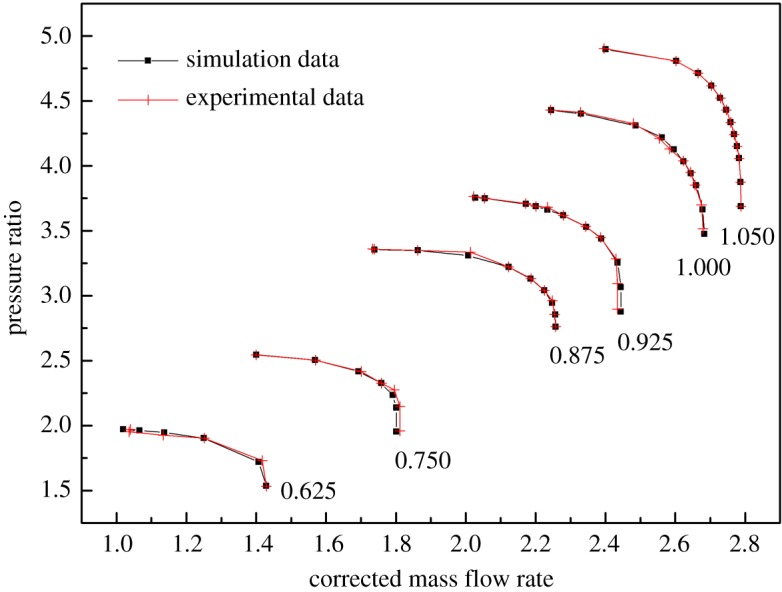
Pressure ratio map of the original compressor with experimental data and simulation data.

**Figure 2.**
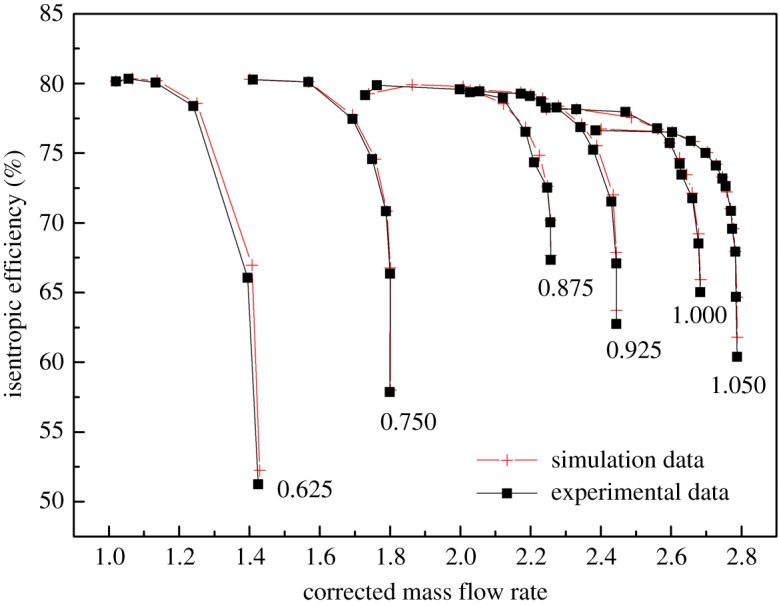
Isentropic efficiency map of the original compressor with experimental data and simulation data.

**Figure 3.**
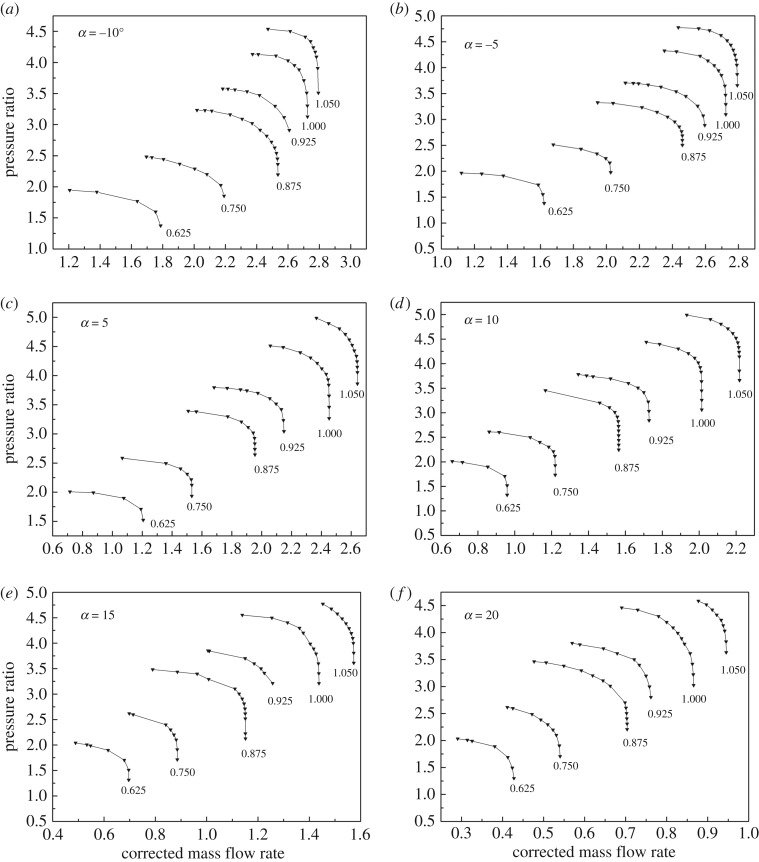
Pressure ratio map of the VGC with different DVAs.

**Figure 4.**
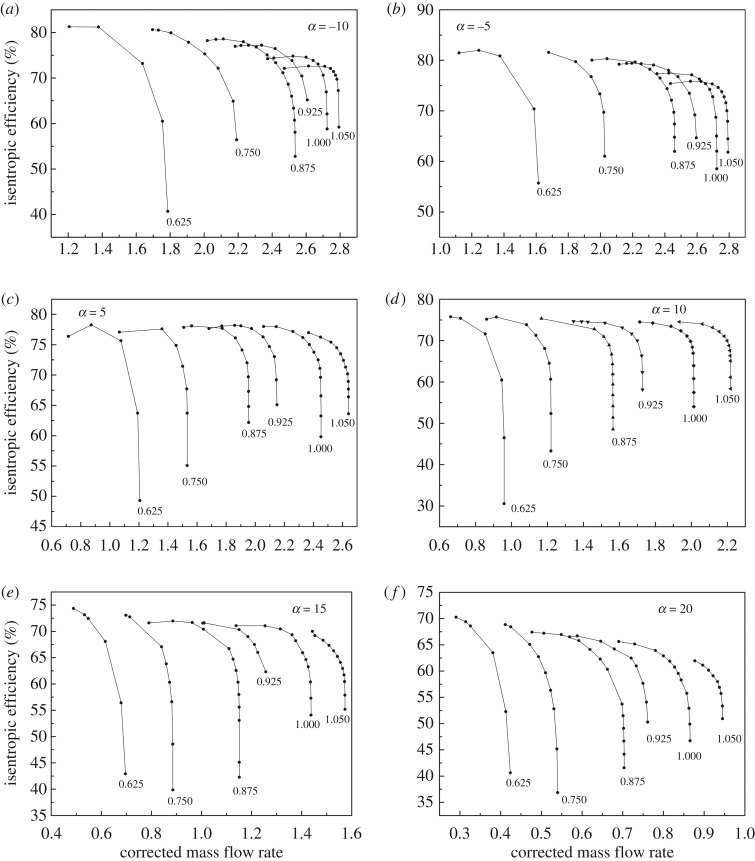
Isentropic efficiency map of the VGC with different DVAs.

As shown in figures 1 and 2, the performance map of the original compressor is obtained from the manufacturer. Many researchers choose CFD instead of the compressor test bench to obtain the performance map of the compressor, because the CFD method reaches an acceptable accuracy as well as time and cost savings with a suitable viscous model. In this paper, in order to obtain the performance of the VGC to validate the proposed method, the CFD model of the original compressor is built first. The simulation data and experimental data are compared in figures 1 and 2. The speed on the speed line is relative speed. It can be seen that the CFD model fit the experimental data accurately, with the root mean square (RMS) less than 3%. Therefore, based on this CFD model, the VGC is introduced by rotating the angle of original compressor diffuser vanes, and its computational result is used as the performance data of the VGC, which is shown in figures 3 and 4. The speed line of different diffuser vane angles is distinguished with different marks. The angles of the diffuser vanes differ from −10° to 20° in which zero means that the performance of the VGC is the same as that of the original compressor, and the flow area of the diffuser increases with decrease in the angle.

### 2.2. The characteristic line-fitting methods

The objective of the method is finding out a single mathematical expression for all available data; it means every speed line has the same form. The accuracy of the fitting methods depends on the quality of the data and the complexity of mathematical expression chosen, etc.

#### 2.2.1. The ellipse equation

After a review of several methods, such as using the look-up table, polynomial and ANN, for presenting the relationship between the pressure ratio $\pi_{C}$, the isentropic efficiency $\eta_{C}$ and the corrected mass flow rate $m_{C}$, the corrected rational speed *N*, the most robust mathematical approach is to use the ellipse equation, where each speed line belongs to an elliptic curve [39]. The equation presents the speed line in the pressure ratio map, which is given by

2.1 $${\left(\frac{m_{c0}-x_{0}}{a_{\pi_{c}}}\right)}^{2}+{\left(\frac{\pi_{c0}-y_{0}}{b_{\pi_{c}}}\right)}^{2}=1,$$

where $a_{\pi_{c}}$ and $b_{\pi_{c}}$ are, respectively, the semi-major and semi-minor axes of the ellipse. In addition, $m_{c0}$ and $\pi_{c0}$ are, respectively, the corrected mass flow rate and the pressure ratio when the centre of the ellipse is fixed at $\left(x_{0},y_{0}\right)$. Considering the rotation of each ellipse, the rotational angle is $\theta_{\pi_{c}}$, the new coordinates of the ellipse$\left(m_{c},\pi_{c}\right)$ are given by

2.2 $$m_{c}=m_{c0}\cos(\theta_{\pi_{c}})-\pi_{c0}\sin(\theta_{\pi_{c}})$$

and

2.3 $$\pi_{c}=m_{c0}\sin(\theta_{\pi_{c}})+\pi_{c0}\cos(\theta_{\pi_{c}}).$$

With the same method, the speed line of the efficiency map is given by

2.4 $${\left(\frac{m_{c0}-x_{0}}{a_{\eta_{c}}}\right)}^{2}+{\left(\frac{\eta_{c0}-y_{0}}{b_{\eta_{c}}}\right)}^{2}=1,$$

where $a_{\eta_{c}}$ and $b_{\eta_{c}}$ are, respectively, the semi-major and semi-minor axes of the ellipse, and $\eta_{c0}$ means the efficiency. Once again, the rotational angle of ellipse $\theta_{\eta_{c}}$ is introduced, and the mass flow rate $m_{c}$ and the efficiency $\eta_{c}$ are, respectively, given by

2.5 $$m_{c}=m_{c0}\cos(\theta_{\eta_{c}})-\eta_{c0}\sin(\theta_{\eta_{c}})$$

and

2.6 $$\eta_{c}=m_{c0}\sin(\theta_{\eta_{c}})+\eta_{c0}\cos(\theta_{\eta_{c}}).$$

The relationship between the pressure ratio and efficiency is established through equations (2.2) and (2.5).

Depending on the complex degree of elliptical function, three approaches have been proposed for fitting the speed lines of the pressure ratio map and the efficiency map, which are as follows:

Approach 1: centre at (0, 0) ($\left(x_{0},0\right)$ for efficiency map) and no rotation.

Approach 2: centre at (0, 0) and rotating the axes with angle θ.

Approach 3: centre at $\left(x_{0},y_{0}\right)$ and rotating the axes with angle θ.

**Figure 5.**
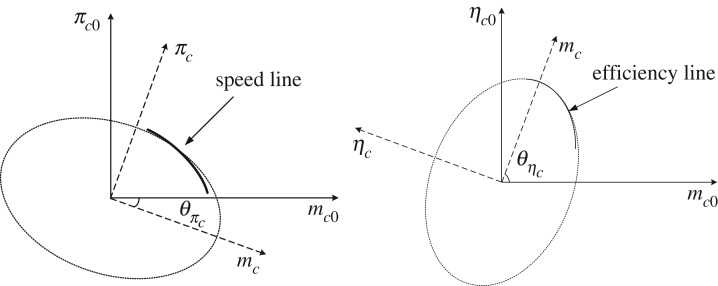
The illustration of elliptical function with rational axes.

As shown in the literature [40], despite approach 1 having the least coefficients, it has the worst prediction accuracy of the speed lines, which is not acceptable. Approach 3 has a high accuracy of prediction, but it has a huge number of coefficients, reaching 100, which is not suitable for fast prediction, with very poor robustness. In this paper, approach 2 is chosen to predict the speed line and because of this, it has a similar accuracy to approach 3; moreover, the number of coefficients decreases greatly, reaching 23 in the literature [40]. The schematic illustration of approach 2 is shown in figure 5.

In approach 2, equations (2.1) and (2.4) are transformed to

2.7 $$\frac{{m_{c}}^{2}}{{a_{\pi c}}^{2}}+\frac{{\pi_{c}}^{2}}{{b_{\pi c}}^{2}}=1$$

and

2.8 $$\frac{{m_{c}}^{2}}{{a_{\eta c}}^{2}}+\frac{{\eta_{c}}^{2}}{{b_{\eta c}}^{2}}=1.$$

According to equations (2.2), (2.3), (2.5), (2.6), (2.7) and (2.8), the uncertain coefficients (i.e. $a_{\pi c}$, $b_{\pi c}$, $\theta_{\pi c}$, $a_{\eta c}$, $b_{\eta c}$ and $\theta_{\eta c}$) at each speed line's ellipse equation are confirmed.

#### 2.2.2. The Levenberg–Marquardt iterative method [41]

Unfortunately, because elliptical equations have strong nonlinearity, the equations cannot be solved by reducing the number of coefficients. Generally, the equation is solved by iterating the equations with a given initial values of coefficients. The Gauss–Newton iterative algorithm is a simple and fast iterative method which uses Taylor series expansion to approximate the nonlinear regression model. However, the iterative accuracy and iterative time of the Gauss–Newton iterative algorithm strongly rely on the initial values. Not only could the iterative time be long, but also the iteration could fail when choosing unsuitable initial values.

To reduce the effect of initial values on the iterative process, the Levenberg–Marquardt iterative method (LM), a damped least square method, is introduced into the iterative process. The LM method is an improvement of the Gauss–Newton iterative algorithm by introducing the damping factor *λ* into the process of iteration. When *λ* equals zero, the LM method degenerates into the Gauss–Newton iterative algorithm. The LM method turns into the steepest descent method when *λ* approaches infinity. The LM method will change the *λ* automatically based on the results of the last iteration. With the automatic change of *λ*, the LM method makes the transition between the Gauss–Newton iterative algorithm and the steepest descent method. Therefore, the LM method combines the advantages of the two methods, i.e. The LM method has both the fast convergence of Gauss–Newton iterative algorithm and the robustness of the steepest descent method.

**Figure 6.**
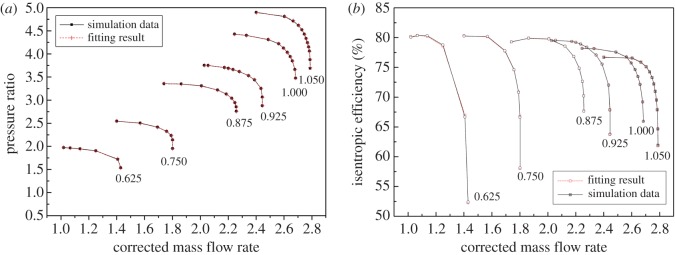
The fitting results of the VGC with elliptical equation and the LM method compared to simulation data.

**Figure 7.**
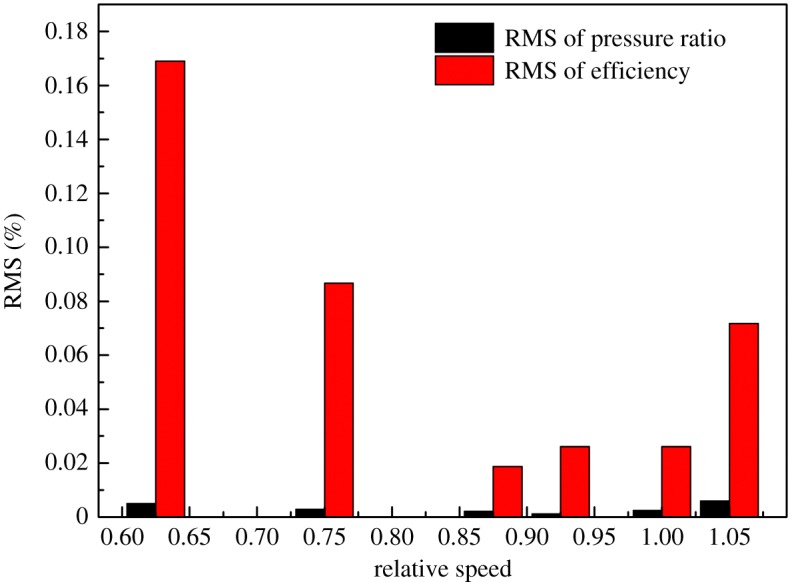
The RMS of the fitting result with elliptical equation and the LM method.

Results of elliptic equation fitting with the LM method are shown in figure 6, in which the DVA equals zero. The RMS defined in equation (2.9) is shown in figure 7.

2.9 $$f_{R}=\sqrt{\frac{\sum_{i=0}^{m}{\left[(z_{ip}-z_{i})\text{/}z_{i}\right]}^{2}}{m}},$$

where *m* is the number of data points of the speed line, *z_i_* denotes the performance data of point *i* and *z_ip_* is the predictive performance data of point *i.*

#### 2.2.3. The partial least-squares methods

Through the ellipse equation and the LM iterative method, the coefficients of each speed line are determined. As for predicting the performance of the VGC, it is inevitable to establish the relationship between different speeds and different vane angles of each coefficient with regard to the ellipse equation. As the DVA is constant, the coefficients can be seen as only the speed-related functions, seen in the following equation:

2.10 f=f(n),

where *n* is the speed of the compressor.

In this situation, the functions (2.10) can be expressed as polynomials, seen in the following equation:

2.11 $$f(n)=f_{k}n^{k}+\cdots +f_{1}n+f_{0},$$

where *k* is the order of the polynomial function.

When the DVA *α* is taken into consideration, equation (2.10) can be transformed to equation (2.12). The sub-coefficients in equation (2.11) can be treated as the polynomial functions of *α*, as shown in equation (2.13) ( *f*_1_ for example). Then equation (2.11) turns into equation (2.14), and equation (2.15) is the final form of equation (2.14).

2.12 f=f(n,α),

where *α* denotes the DVA.

2.13 $$f_{1}(\alpha )=f_{1p}\alpha^{p}+\cdots +f_{11}\alpha +f_{10},$$

where *p* denotes the highest order of the polynomial.

2.14 $$f=\sum_{i=0}^{k}\left(\sum_{j=0}^{p}f_{ij}\alpha^{j}\right)n^{i}$$

and

2.15 $$f=\sum_{i=0}^{k}\sum_{j=0}^{p}f_{ij}n^{i}\alpha^{j}.$$

In equation (2.15), the highest order of the polynomial is *k* *+* *p*, and the number of sub-coefficients is (*k* *+* 1)(*p* *+* 1). With the introduction of the DVA, the function of coefficients becomes complicated, and the partial least-squares method (PLS) is introduced to solve this complicated problem. The PLS regression method is a novel multiple statistical analysis method developed from the field of chemistry, which was proposed by Wold in 1983 [42]. PLS combines the basic functions of multiple linear regression analysis, canonical correlation analysis and principal component analysis together, and integrates the data analysis method of the modelling prediction type with the non-model-based data analysis method organically [25]. The detailed information of PLS can be found in the literature [35].

**Figure 8.**
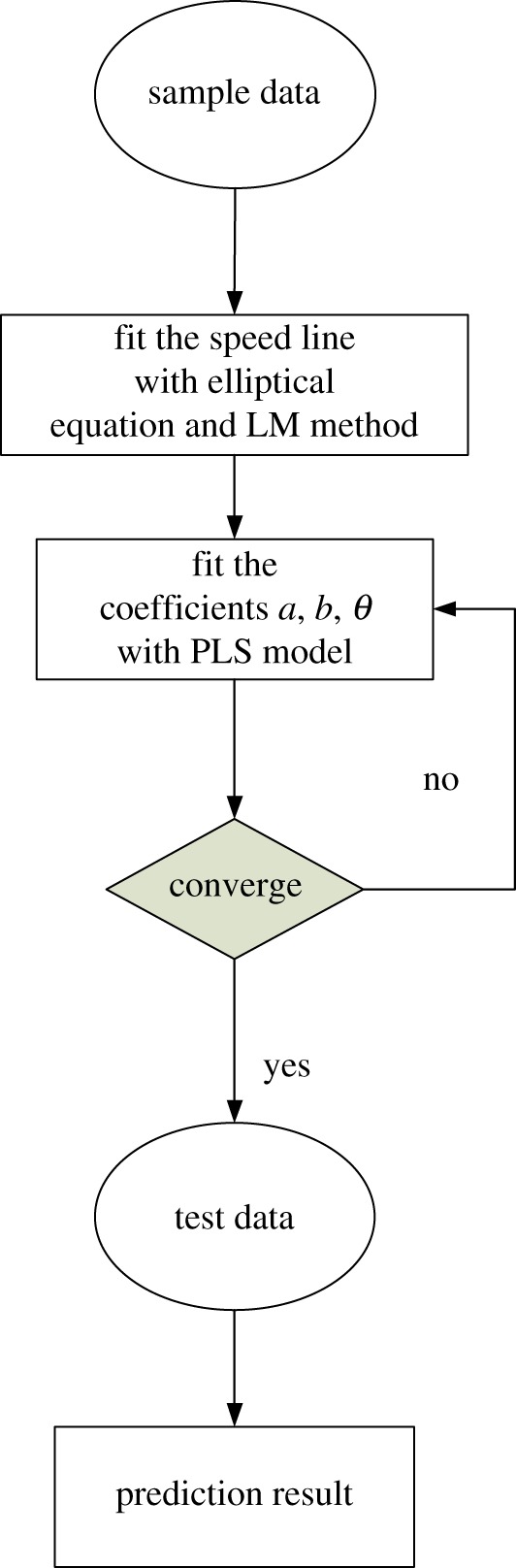
The flow chart of the prediction model.

The flow chart of the proposed prediction model is shown in figure 8. Firstly, the speed lines of sample data are fitted by the elliptical equation and the LM method. Then the coefficients of elliptical equation, *a*, *b* and *θ* are introduced in the PLS model to establish the relationship between the coefficients, speed and DVA, adjusting the maximum order of the polynomial to compromise the fitting accuracy of coefficients and the number of sub-coefficients. Once the PLS model is confirmed by the former steps, the speed and the DVA of test data are input to the PLS model to obtain the coefficients of the corresponding speed line. Finally, according to the coefficients, elliptical equation and corresponding corrected flow rate, the prediction results are obtained.

To verify the effectiveness of the proposed methods, the BPNN and look-up table methods are compared with the proposed method. The detailed information about the look-up table and BPNN methods can be found in [21] and [28], respectively.

## 3. Application and analysis

To build a VGC performance prediction model, the performance data of the VGC are divided into two parts: sample data and test data. The sample data are used to build the prediction model and determine the sub-coefficients. The test data, used to verify the accuracy and effectiveness of the predictive model, include an entire performance map with the DVA equal to 5 for interpolation, and the performance data of minimum speed for extrapolation with other DVAs. The test data are also used to compare the proposed method and other methods.

### 3.1. Fitting performance characteristic lines with elliptical equation and LM

**Figure 9.**
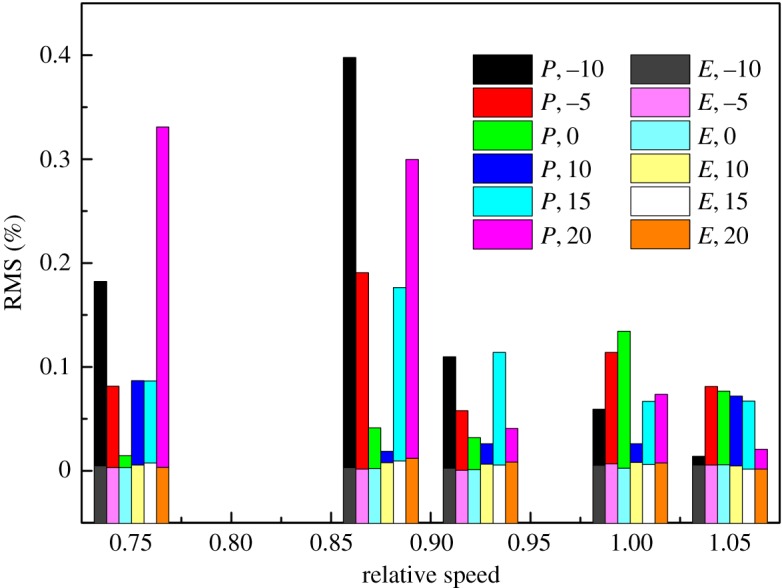
The RMS of fitting methods with sample data.

To demonstrate the effectiveness of the elliptical equation with the LM method and obtain the coefficients of each elliptical equation of the speed line, the speed lines of the sample data were fitted with the elliptical equation, and the LM method was used in the iteration process. The result of the fitting process is shown in figure 6 with the DVA equal to zero, for example. And the RMS under each diffuser vane and speed is shown in figure 9, where *P* denotes the RMS of the pressure ratio, *E* the RMS of the isentropic efficiency, and the number behind the *P* or *E* is the angle of the diffuser vanes. In figure 6, it can be seen that the error between the simulation data and the fitting result is so small that there is no visual difference. The detailed difference between the simulation data and the fitting result is shown in figure 7. Figure 7 illustrates that the RMS of the pressure ratio is less than 0.01%, and the RMS of the isentropic efficiency increases greater than the RMS of the pressure ratio but does not exceed 0.2%; this trend is also shown in figure 9, which is the RMS of the pressure ratio and the isentropic efficiency with all sample data. In figure 9, the RMS of the pressure ratio is less than 0.01% except for the condition that the relative speed equals 0.875 and the DVA equals 20. The RMS of the isentropic efficiency does not exceed 0.4%. In figures 6, 7 and 9, it is obvious that the elliptical equation and the LM method can well fit the sample data with high accuracy, which provides a support for predicting the test data.

### 3.2. Fitting the coefficients of elliptical equations with PLS methods

**Figure 10.**
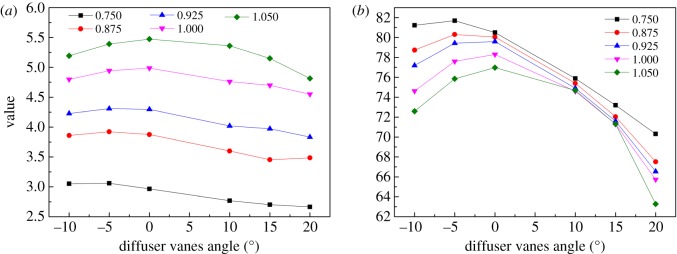
(*a,b*) The coefficient *a* of pressure ratio and efficiency varies with the relative speed and the DVA.

**Figure 11.**
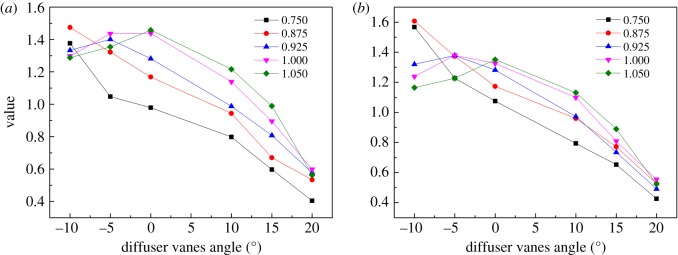
(*a,b*) The coefficient *b* of pressure ratio and efficiency varies with the relative speed and the DVA.

**Figure 12.**
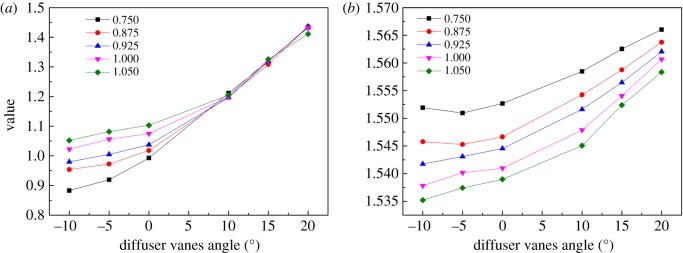
(*a,b*) The coefficient *θ* of pressure ratio and efficiency varies with the relative speed and the DVA.

**Figure 13.**
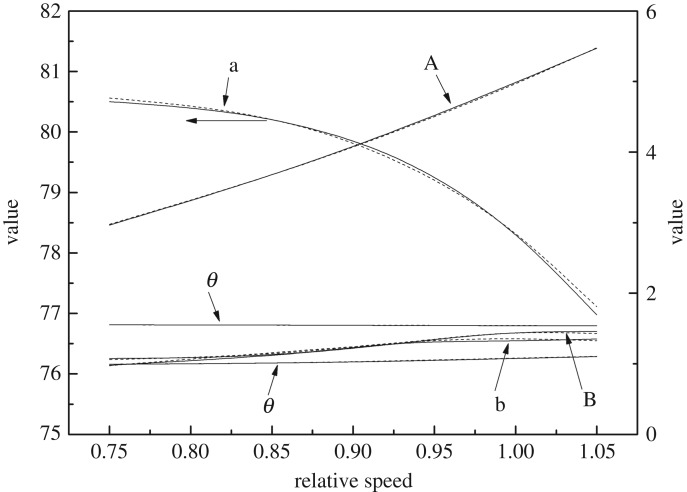
The comparison of the fitting result and coefficients.

Owing to the endeavour of the previous steps, the coefficients of the elliptical equation are confirmed, as shown in figures 10–12 for *a, b* and *θ,* respectively. The sub-figure marked (*a*) in each figure is the coefficient of the pressure ratio map, and the others are the coefficients of the efficiency map. The relative speed of each line is marked in each sub-figure. Then the coefficients are input into the PLS model as the dependent variables to establish the relationship between the coefficients and the relative speed, the DVA. To observe the influence of the maximum order of relative speed and DVA on the coefficients and the performance of the VGC, the maximum order of relative speed and DVA is set to 3 and 4, respectively. Table 1 shows the influence of the maximum order on the coefficients. From table 1, it can be seen that the PLS model has the worst fitting accuracy but has the least number of sub-coefficients, reaching 96 when the maximum orders are 3 and 3, and has the best fitting accuracy with the highest number of sub-coefficients reaching 150 when the maximum orders are 4 and 4. Fortunately, the RMS of *a* and *θ* do not exceed 1% despite the maximum order, in the pressure ratio map and the efficiency map. But the RMS of *b* reaches 2.87% and 2.74% for the pressure ratio map and the efficiency map, respectively, when the maximum orders are 3 and 3. To fit the coefficient *b* accurately, the maximum orders are fixed at 4 and 4, whose RMS is 1.43% and 1.42%, respectively, and maximum orders of 3 and 3 are chosen to fit the coefficients *a* and *θ.* In this situation, the sub-coefficients decrease from 150 to 114 and the fitting accuracy is acceptable. Figure 13 depicts the fitting result with the PLS model and the coefficients of the elliptical equation, with the DVA equalling 0 as an example. In figure 13, solid lines depict the coefficients obtained by elliptical equations and the dash lines represent the fitting result with the PLS model. The upper-case letters indicate the corresponding coefficients of the pressure ratio map and lower-case letters the corresponding coefficients of the efficiency map.

**Table 1.** The influence of the maximum order on coefficient evaluation.

maximum order of relative speed and DVA (°)	total number of sub-coefficients	total RMS of *a, b* and *θ* for pressure ratio map and efficiency map, respectively (%)
4, 4	25 × 3 × 2	0.28, 1.43, 0.23; 0.12, 1.42, 0.01
4, 3	20 × 3 × 2	0.4, 2.78, 0.56; 0.28, 1.46, 0.02
3, 4	20 × 3 × 2	0.47, 1.87, 0.24; 0.18, 2.72, 0.015
3, 3	16 × 3 × 2	0.53, 2.87, 0.56; 0.31, 2.74, 0.024

### 3.3. Validations and prediction of VGC prediction model

The maximum order and the sub-coefficients were determined in §3.2; then the coefficients *a, b* and *θ* are expressed as follows:

3.1 $$\begin{array}{r} a_{\pi c}=\sum_{i=0}^{3}\sum_{j=0}^{3}a_{\pi ij}n^{i}\alpha^{j}, \end{array}$$

3.2 $$\begin{array}{r} b_{\pi c}=\sum_{i=0}^{4}\sum_{j=0}^{4}b_{\pi ij}n^{i}\alpha^{j}, \end{array}$$

3.3 $$\begin{array}{r} \theta_{\pi c}=\sum_{i=0}^{3}\sum_{j=0}^{3}\theta_{\pi ij}n^{i}\alpha^{j}, \end{array}$$

3.4 $$\begin{array}{r} a_{\eta c}=\sum_{i=0}^{3}\sum_{j=0}^{3}a_{\eta ij}n^{i}\alpha^{j}, \end{array}$$

3.5 $$\begin{array}{r} b_{\eta c}=\sum_{i=0}^{4}\sum_{j=0}^{4}b_{\eta ij}n^{i}\alpha^{j} \end{array}$$

3.6 $$\begin{array}{r} \;\mathrm{and}\;\theta_{\eta c}=\sum_{i=0}^{3}\sum_{j=0}^{3}\theta_{\eta ij}n^{i}\alpha^{j}, \end{array}$$

where $a_{\pi ij}$, $b_{\pi ij}$, $\theta_{\pi ij}$, $a_{\eta ij}$, $b_{\eta ij}$, $\theta_{\eta ij}$ are the sub-coefficients of each equation, respectively.

**Figure 14.**
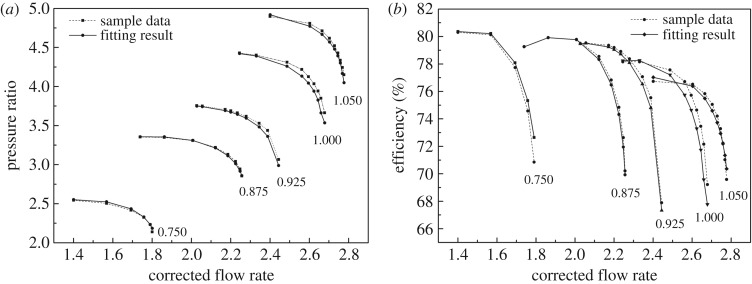
(*a,b*) The VGC performance fitting result when the DVA equals 0.

According to equations (3.1)–(3.6), the coefficients *a, b* and *θ* are confirmed with a certain relative speed and certain DVA. The elliptical function, therefore, is confirmed to predict the performance of the VGC. Figure 14 depicts the VGC performance so that the DVA equals 0. Figure 14 and table 2 demonstrate the accuracy of the prediction model with the maximum RMS of each speed line less than 2%, both in the pressure ratio and efficiency.

**Table 2.** The RMS of the fitting result.

relative speed	RMS of pressure ratio (%)	RMS of efficiency (%)
0.75	1.009	1.128
0.875	0.530	0.480
0.925	1.249	0.512
1	1.975	1.819
1.05	1.262	0.561

**Figure 15.**
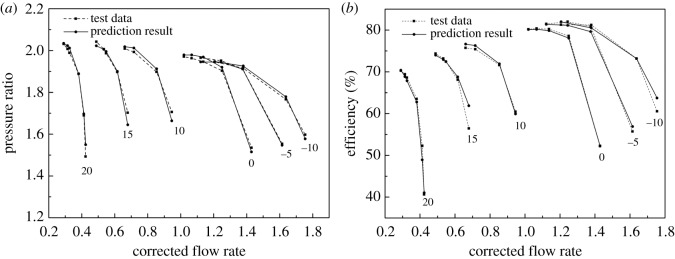
(*a,b*) The prediction result of VGC performance with different DVAs.

**Figure 16.**
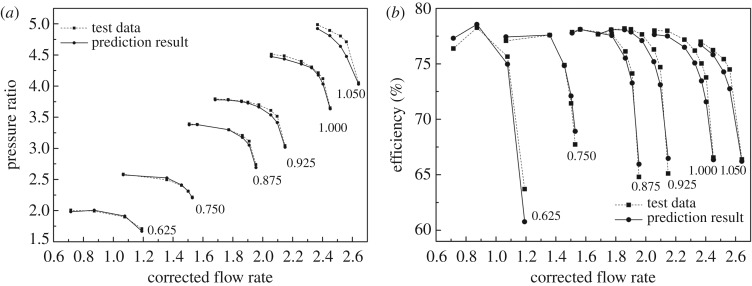
(*a,b*) The prediction result of VGC performance when the DVA equals 5.

Figure 15 shows the predictive result compared to test data with the minimum relative speed, which equals 0.625. Figure 16 shows the prediction performance compared to test data when the DVA equals 5. The RMS is given in table 3.

**Table 3.** The RMS of prediction results.

relative speed and DVA	RMS of pressure ratio %	RMS of efficiency %
0.625, −10	0.6884193	2.7100514
0.625, −5	0.5819134	1.4180841
0.625, 0	0.9648913	0.3787116
0.625, 5	1.4630324	2.4352065
0.75, 5	0.6360919	0.9145616
0.875, 5	1.1397356	0.9292457
0.925, 5	1.3308175	1.2092946
1, 5	1.0812276	1.5520649
1.05, 5	2.8911417	1.2910451
0.625, 10	1.4035895	0.9747743
0.625, 15	1.5848963	4.3589366
0.625, 20	1.6599611	2.7637363

From figures 15 and 16, the prediction model shows a favourable prediction accuracy either with the minimum relative speed or with fixed DVA with the RMS less than 5%, and only 5 prediction points exceed 2%. The maximum RMS occurs for efficiency prediction when the relative speed equals 0.625 and the DVA equals 15 simultaneously. The RMS of efficiency reaches 4.4% nearly, because the efficiency is strongly sensitive to the corrected flow rate when the operation point is close to choking, i.e. a tiny perturbation in the corrected flow rate will result in a drastic change in efficiency.

### 3.4. Compared with BPNN and look-up table method

**Figure 17.**
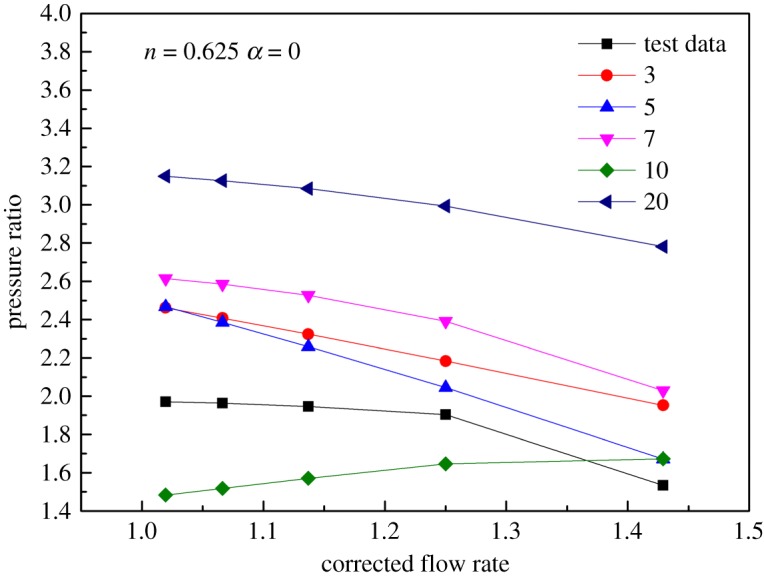
The influence of different hidden layers on the prediction result.

**Figure 18.**
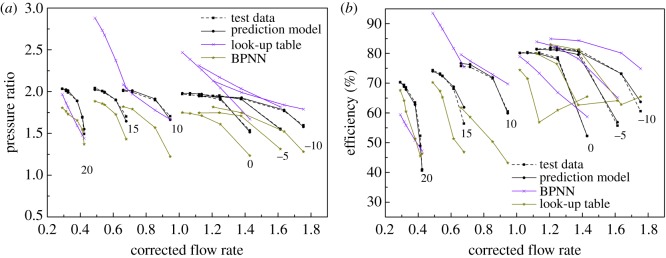
(*a,b*) Comparison of the prediction results when the relative speed equals 0.625.

**Figure 19.**
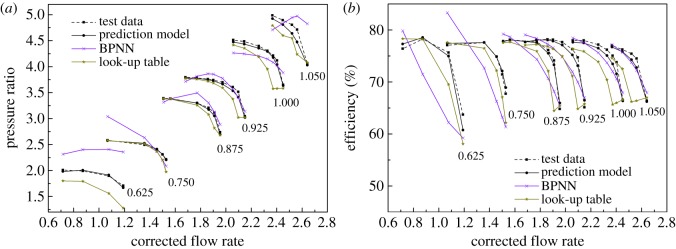
(*a,b*) Comparison of the prediction results when the DVA equals 5.

To illustrate the accuracy of the prediction model, the BPNN and look-up table methods are introduced for comparison, which is comprehensively used in modelling. The look-up table interpolates and extrapolates sample data linearly. To determine the hidden layers of the BPNN, the prediction results with different hidden layers of the BPNN are researched, which are shown in figure 17. It is obvious that the prediction accuracy is best when the hidden layers equal 5; then the hidden layer is determined as 5. Figures 18 and 19 show the comparison of these three methods with test data. It is obvious that the proposed prediction model has apparently better performance than the look-up table and BPNN methods not only in quantitative prediction but also in qualitative prediction. In figure 18, the relative speed equals 0.625 as extrapolation, and the prediction results of the BPNN and look-up table methods depict unacceptable prediction ability either in the pressure ratio map or in the efficiency map, even though the look-up table has the ability to predict the tendency of the speed lines in the pressure ratio map. In figure 19, the DVA equals 5 as interpolation, and the look-up table and BPNN methods reveal the ability of tendency prediction to some degree (look-up table at low relative speed and BPNN at high relative speed). But the quantitative prediction results of the look-up table and BPNN methods are rather worse than those of the proposed prediction model. The difference between the look-up table, BPNN and the test data is really great and can be easily observed in figure 19.

**Figure 20.**
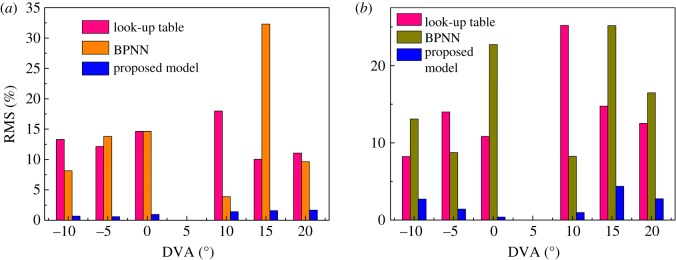
(*a,b*) The RMS of different prediction models with different DVAs.

**Figure 21.**
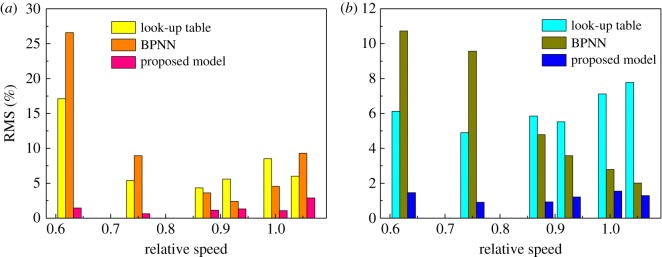
(*a,b*) The RMS of different prediction models with different relative speeds.

To illustrate the prediction accuracy of the proposed model, the RMS of the pressure ratio and the efficiency under different prediction models are listed in tables 4 and 5, which represent different DVAs with relative speed equal to 0.625 and different relative speeds with the DVA equal to 5°, respectively. Figures 20 and 21 are the column charts of the tables 4 and 5, respectively. In figure 20, the RMS of the proposed prediction model is far less than those of the look-up table and BPNN method both in pressure ratio prediction and in efficiency prediction. The RMS of the look-up table is similar to that of BPNN, which means that the prediction ability of the BPNN method is not stronger than that of the look-up table method. In figure 21, the RMS of the proposed prediction model is still less than those of the look-up table and BPNN methods both in pressure ratio prediction and in efficiency prediction, but the gap of RMS between the three methods is reduced. It is shown that the look-up table and BPNN methods have better prediction ability in interpolation than in extrapolation.

**Table 4.** The RMS of the prediction model with the relative speed equal to 0.625.

	RMS of look-up table %		RMS of BPNN %		RMS of the proposed model %	
DVA	pressure ratio	efficiency	pressure ratio	efficiency	pressure ratio	efficiency
−10	13.32	8.23	8.14	13.09	0.69	2.71
−5	12.13	14.00	13.80	8.75	0.58	1.42
0	14.64	10.84	14.64	22.99	0.96	0.38
10	18.00	25.20	3.88	8.26	1.40	0.97
15	10.03	14.76	32.30	25.18	1.58	4.36
20	11.05	12.51	9.64	16.48	1.66	2.76

**Table 5.** The RMS of the prediction model with the DVA equal to 5.

	RMS of the look-up table %		RMS of the BPNN %		RMS of the proposed model %	
relative speed	pressure ratio	efficiency	pressure ratio	efficiency	pressure ratio	efficiency
0.625	17.10	6.12	26.59	10.73	1.46	1.46
0.75	5.39	4.90	8.95	9.57	0.64	0.91
0.875	4.34	5.85	3.62	4.79	1.14	0.93
0.925	5.61	5.52	2.41	3.58	1.33	1.21
1	8.51	7.12	4.56	2.79	1.08	1.55
1.05	6.00	7.78	9.31	2.01	2.89	1.29

According to the analysis and comparison mentioned above, the proposed prediction model is more suitable than the look-up table and BPNN methods for predicting VGC performance both in interpolation and extrapolation.

## 4. Conclusion and discussions

A prediction model based on the elliptical equation and the PLS model was proposed to predict VGC performance. The prediction model was validated with the sample data and experimental data. The prediction accuracy of the look-up table, BPNN and proposed prediction model was compared against the test data. Some meaningful conclusions are obtained as follows:

1. The elliptical equation with a fixed centre and rational axes can accurately fit the speed lines of the VGC both in the pressure ratio map and the efficiency map.
2. The connection of the elliptical equation with the PLS model not only establishes the relationship between the coefficients and the relative speed and DVA, but also reduces the difficulty of system modelling.
3. By choosing the suitable maximal order of the polynomial with regard to coefficients, the prediction accuracy of the prediction model and the number of sub-coefficients can be compromised comfortably.
4. The proposed model shows acceptable prediction accuracy, with the maximum RMS less than 5%, both in the minimum relative speed line with extrapolation, and the entire performance map when the DVA equals 5 with interpolation.
5. The prediction accuracy of the proposed prediction model is much better than that of the look-up table method and the BPNN method.
6. The proposed prediction model can be expected to improve the accuracy of the thermodynamic performance model of a diesel engine.

## Data accessibility

Our primary data can be found at the website: http://www.jtp.com.cn/cpzx/zyq/jthxl/223820.htm. The other data and calculation results can be found at Dryad: http://dx.doi.org/10.5061/dryad.p4c2v [43].
